# Mr. Shillito, on Intermittents

**Published:** 1805-08-01

**Authors:** Charles Shillito

**Affiliations:** Member of the Royal College of Surgeons, and Surgeon to the West Essex Militia. Colwell Baracks, Isle of Wight


### *To the Editors of the Medical and Physical Journal.*

GENTLEMEN,

MUCH difference of opinion having existed respecting the utility of the different mineral tonics in the cure of Intermittents, I have been induced to note down some observations on their exhibition, as also, on the cure of ague in general, which I have been enabled to make, from the circumstance of having had from fifty to sixty cases of that disease in my hospital within the last four months; and also, in the same period, an equal if not greater number under my care, that have occurred among the women and children allied to the regiment to which I have the honor to belong. I have taken the liberty of transmitting them for your perusal; and if they should be thought sufficiently interesting, you are at liberty to insert them in your valuable Journal.

A succession of agues among the privates of the West Essex Militia first occurred in the beginning of March; the regiment then being in garrison at Sheerness, at which place the disease was most decidedly endemial; from that period until the 24th of April, when it arrived in the Isle of Wight, about thirty men, women, or children, were taken ill with the disease; and from its arrival here, until within the last three weeks, scarce a day passed without instances of fresh attacks. Not to enter fully into

 the the predisposing or proximate causes, or indulge in any speculative theory, I cannot help saying, that from the salubrious air, and elevated situation of this country, together with the unfrequency of ague occurring among the natives,* I cannot conceive the cause to have originated here, but that the evil must for some time have laid dormant. The debilitating effects of so many inhabitants having been crowded together in the garrison at Sheerness; the formation of the barracks ill adapted for the purpose of ventilation; in addition to the low and marshy country adjoining, must, I should suppose, have produced a predisposition to ague in the system, which may have been afterwards called into action by cold, or any casual cause of obstructed or diminished perspiration. At the commencement of the disease, where the biliary system was at all deranged, an emetic, or calomel and rhubarb was administered; after which the solutio arsenici was uniformly given, and with the greatest success imaginable, so far, as it prevented a recurrence of the fit, and paved the way for the bark, or some other vegetable tonic to effect a compleat cure. However serviceable it may be in preventing for a time a return of the paroxysm, I am convinced by experience, whoever trusts to it alone, to reinstate his patient in perfect health and strength, will in many instances indeed be grievously disappointed; having constantly observed, where it has not been discontinued after a remission of the fit, and the bark or some other tonic substituted, the same swelling of the legs, the same pallid appearance of the lips, and the same want of tone in the system in general, have continued until between the seventh and eleventh day, when a return of the disease has been but too often the unpleasing event; on the contrary, where the solution has been discontinued, and the bark given, scarce ever have I known it fail to render the cure compleat; in cases where there has been a disposition to nauseate the bark, I have found the quassia after the exhibition of the solution of the greatest utility.

Supposing the arsenic to procure a remission of the attack by any tonic powers, as a tonic it has certainly its "ne plus ultra," and after a certain time, instead of strength-

---

* Since writing the above I have had some conversation with a very intelligent practitioner, who informs me, ague is by no means a rare occurrence in this part of the island, although he knows of no recent instances, nor upon the most minute enquiry have I been able to hear of more than two or three cases having lately occurred.

strengthening, most undoubtedly debilitates the stomach and system in general; never have I seen its use persevered in, (whether successful or not in the first instance) more than ten or twelve days, without its ultimately proving prejudicial. Both in dose and preparation I have used it according to the formula given by Dr. Fowler, and have never observed it produce any irritation in the stomach or intestines, but what (omitting its exhibition) a few drops of laudanum have immediately composed. I am well aware, from the mildness of the attack, and season of the year, that many of the cases might have recovered without that or any other medicine; but from so constantly having observed its gradual and unvarying effects, I must be an errant Cynic, indeed, for one moment to be insensible to its value; two cases however occurred, in which the solution proved inefficacious; the bark liberally given unavailing, and which run the gauntlet through the other various tonics, both vegetable and mineral, without any good effect; and which also derived no benefit from that dernier resort, "a change of air," although it was experienced in no small degree, as a removal from the marshes in the isle of Sheppy to the more bracing air in the Isle of Wight must evince. From its having been recommended from the most respectable quarter, I was induced to try how far the shock from a shower-bath, or a sudden immersion in salt water upon the approaching paroxysm, might be able to avert it; from our contiguous situation to the sea, I was inclined to make essay of the latter; the gradual declivity of the shore in this bay, rendered some assistance necessary, to make the immersion general and instantaneous; and which I was enabled to accomplish by the assistance of four men, who sustained my patient in a blanket above the surface of the water, until there was sufficient depth, when he was suddenly immersed, immediately brought on shore, and rubbed dry with a coarse cloth; the essential utility of which far exceeded my most sanguine expectations, as the two following cases will prove.

John Spencer, of the grenadier company, aged twenty-four, a strong athletic man, was taken ill with an ague of a tertian type, at Sheerness, towards the end of April, which continued invariably recurring every second day, until the 1st of June, when having discontinued all medicines, at nine A. M. (about half an hour before the usual return of the attack) he was immersed in the sea; no cold fit followed; a slight accession of fever only took place.

3d. and 5th. The immersion repeated at the same hour, a slight accession of fever still following.

7th. Immersion repeated; no fever whatever; was ordered to bathe every morning, and allowed a pint of porter daily; no medicines whatever were exhibited.

17th. Was discharged for partial duty. Has now taken general duty a fortnight, and, to use his own emphatic words, "was never heartier in his life."

John Hills, a thin puny lad, aged nineteen, was admitted into the hospital at Sheerness on the 21st of April, with an intermittent of a quotidian type, which in the course of a few days degenerated into a tertian; had taken the bark, sol. arsenici, zinc. vitriol. &c. &c. without any good effect.

June 7. The fit as usual, hot paroxysm lasting several hours, legs much swelled, and much general debility from the repeated attacks; was ordered to discontinue his medicines.

11th. Seven A. M. (the fit usually returning soon after) he was immersed in the sea; at nine A. M. he experienced a slight chilly sensation, but no direct shaking; a little fever took place, but which lasted not more than an hour.

13th. Immersion repeated; no shivering or fever followed; was ordered to bathe every morning, and allowed a generous diet. He gradually recovered his strength, and was on the point of being discharged the hospital, when he was again attacked; the immersion was again had recourse to with the same success, and on the 12th instant he was discharged quite well.

The exhibition of opium previous to the return of the paroxysm, I have never found of any essential utility; that it lessens the cold fit is true, but it is only to lengthen, and make more formidable the accession of fever; never have I found it upon repetition prove ultimately successful.

Both the cuprum vitriolatum and zincum vitriolatum, I have repeatedly tried, and have sometimes found them serviceable, but much more frequently totally inefficacious. From the latter having been honoured with the term "specific," much acrimonious controversy has lately taken place; that it is entitled to that honour I can by no means allow, from having repeatedly witnessed its inutility. If I thought it deserving it, I would most readily grant it, as I must confess I cannot see any thing so formidable or terrific in the epithet abstractedly "in se."

*Colwell Baracks, Isle of Wight,* I am, &c.

*July* 14, 1805.

CHARLES SHILLITO,
Member of the Royal College of Surgeons,
and Surgeon to the West Essex Militia.

